# Real-Time Visualization and Quantitation of Vascular Permeability *In Vivo*: Implications for Drug Delivery

**DOI:** 10.1371/journal.pone.0033760

**Published:** 2012-03-29

**Authors:** Desmond B. S. Pink, Wendy Schulte, Missag H. Parseghian, Andries Zijlstra, John D. Lewis

**Affiliations:** 1 Innovascreen, Inc., Halifax, Nova Scotia, Canada; 2 Stonsa Biopharm Inc., Carlsbad, California, United States of America; 3 Department of Pathology, Vanderbilt University, Nashville, Tennesee, United States of America; 4 Department of Oncology, University of Alberta, Edmonton, Alberta, Canada; National Cancer Institute, United States of America

## Abstract

The leaky, heterogeneous vasculature of human tumors prevents the even distribution of systemic drugs within cancer tissues. However, techniques for studying vascular delivery systems *in vivo* often require complex mammalian models and time-consuming, surgical protocols. The developing chicken embryo is a well-established model for human cancer that is easily accessible for tumor imaging. To assess this model for the *in vivo* analysis of tumor permeability, human tumors were grown on the chorioallantoic membrane (CAM), a thin vascular membrane which overlays the growing chick embryo. The real-time movement of small fluorescent dextrans through the tumor vasculature and surrounding tissues were used to measure vascular leak within tumor xenografts. Dextran extravasation within tumor sites was selectively enhanced an interleukin-2 (IL-2) peptide fragment or vascular endothelial growth factor (VEGF). VEGF treatment increased vascular leak in the tumor core relative to surrounding normal tissue and increased doxorubicin uptake in human tumor xenografts. This new system easily visualizes vascular permeability changes *in vivo* and suggests that vascular permeability may be manipulated to improve chemotherapeutic targeting to tumors.

## Introduction

Tumors develop a chaotic vascular network characterized by variable blood pressure and vascular permeability that inhibits effective drug delivery [1]. Many areas within tumors contain irregular blood vessels that are leaky and allow influx of circulating blood components. Sporadic high cell density within the tumor prevents normal tissue drainage [2]. This promotes the accumulation of cellular and blood proteins in the interstitial space, leading to high interstitial oncotic pressure, which inhibits the extravasation of systemic drugs [3]. Ultimately the distribution of systemically circulating drugs in tumors can be unpredictable and irregular since it depends heavily on the passive extravasation of the drug from the vasculature into target tissues [2], [4], [5], [6].

By transiently altering tumor blood vessel physiology during systemic anti-cancer treatment, tissue perfusion and drainage can be enhanced, thereby relieving interstitial hypertension [7], [8]. Prolonged treatment with anti-angiogenic drugs, such as Sunitinib or DC101, normalizes blood flow through the remaining stabilized vasculature. These treatments can improve tumor micro-hemodynamics and effectively lower the interstitial pressure. Consequently, the efficacy of concomitantly or subsequently administered drugs is enhanced due to improved vascular delivery [7], [9], [10]. Similarly, treatment of hepatic tumors with interferon-β (IFN-β) induces tumor vessel maturation and tissue perfusion, which improves delivery of additional therapeutics [11]. Altering oncogenic signaling in tumors can also be used to change their blood-flow dynamics [12]. Specifically, inhibition of the PI3K pathway increases tumor perfusion and simultaneously enhances doxorubicin delivery [13]. These findings indicate that the strategic use of adjuvants to transiently modify tumor blood flow and hemodynamics can facilitate drug delivery to cancer sites.

Normalizing blood flow promotes drug delivery by reducing the interstitial pressure that counteracts diffusion. However, normalizing agents can also reduce vascular permeability. Vascular permeability greatly influences the extravasation of drugs associated with carriers, including liposomes, micelles or other nanoparticles [14], [15], [16]. Recent advances to manipulate vascular permeability exemplify how adjuvant therapy might facilitate the targeting of future and existing anti-cancer therapies to tumor tissues [6], [17], . Unfortunately, the lack of accurate means to quantify vascular permeability is a significant hurdle to predicting its direct influence on drug localization and uptake *in vivo*.

Classically, vascular permeability has been measured using the Miles Assay [19]. This assay determines the leakage of a visible dye from the vasculature into the surrounding tissue spectrophotometrically, with the relative vascular permeability determined as the ratio of extravasated versus intravascular dye. This assay has several limitations, however, that preclude its use in many cases. It is limited to the analysis of a single time-point, which must be selected empirically from pilot experiments. Furthermore, due to the wide range of experimental approaches described in the literature, results are subject to a high degree of variability and their repeatability must be considered. Variability can be mitigated somewhat by using large tissue volumes. Consequently, these experiments are generally performed in rodent models with large group sizes [19], which is both expensive and time-consuming. As the Miles assay is limited to the determination of average permeability over an entire tissue, localized differences in vascular permeability, particularly within tumors, cannot be detected. A dynamic measure of vascular permeability would allow for the assessment of the impact of regional and temporal changes in vascular permeability on drug distribution within solid tumors.

Here, we present an integrated method to visualize and quantify the real-time dynamics of dextrans in a shell-less chick chorioallantoic (CAM) model. Regional and temporal differences in vessel permeability within the tumor microenvironment are captured at high resolution using an intravital imaging approach. The use of dextrans of different molecular weights allows for the concurrent evaluation of vascular permeability and vascular structural integrity. The dynamics of anti-cancer drugs as they move through the vasculature and into tumor tissues can be mimicked with dextrans [20]. Dextrans of various molecular weights can mimic the diffusion of various sized macromolecules including macro-molecular drug carriers (∼70 kDa) and antibodies (∼150 kDa) into the tumor interstitial space. Large dextrans of ∼2000 kDa, are sequestered within the lumen of the tumor vasculature [20]. This work builds upon earlier observations in the shell-less chicken embryo model, which examined microvascular perselectivity during normal angiogenesis in the early stages of CAM development [21]. These authors demonstrated a rapid reduction in microvascular permeability to FITC-dextrans of varying sizes (20–150 kDa) between days 4.5–5.5 of the normal 21-day gestation. They also demonstrated that dextran size correlated with permeability (dextran-20>dextran-40>dextran-70>dextran-150 kDa). Furthermore, while these authors report tumor permeability values for 70 kDa and 150 kDa dextrans, they did not examine it in the shell-less or *ex ovo* chick model. The leakage of small versus large molecular weight dextrans from the vasculature in this model may provide a high-resolution measure of vascular permeability predictive of drug localization *in vivo*.

The CAM is a thin, respiratory tissue for the developing chick embryo characterized by a dense, highly organized network of blood vessels [22], [23]. The physiological responses of the CAM are consistent with those of mammalian tissues [24], [25] and it has provided a physiologically relevant setting for angiogenesis research for more than a century [26], [27], [28], [29], [30], [31]. The commercial availability of fertilized eggs, the ease of embryo culture, and the robustness of the CAM model facilitate large, statistically powerful studies and make it suitable for high throughput approaches. The CAM is not fully immunocompetent in the early embryo [32], and it supports the growth of human and murine tumor xenografts [33], [34], [35]. In addition, in the *ex ovo* model, the CAM is directly accessible for experimental manipulation and imaging. Paired with a fluorescence microscopy platform, this model is well-suited for analyzing drug-induced changes in vascular permeability in tumor xenografts and their microenvironment.

We demonstrate, using this intravital imaging approach, that vascular permeability can be manipulated to modulate the extravasation of small molecules into the local tumor microenvironment. Treatment with vascular endothelial growth factor (VEGF) or a permeability enhancing peptide (PEP) fragment of IL-2 [36] either locally or systemically results in a temporary enhancement of vascular permeability that can be precisely monitored over time. We show that this transient increase in vascular permeability can be exploited to significantly enhance the accumulation of a chemotherapeutic drug within the tumor.

## Methods

### *In vivo* detection of vascular leak

A modified Miles assay [19] was adapted for the CAM model. Chicken embryos (day 15) were injected intravenously with phosphate buffered saline (PBS) recombinant human VEGF_121_ (40 ng, Peprotech) or PEP (0.1 nM, Peregrine Pharmaceuticals) in 50 µL volumes. For local applications, reagents were applied to the CAM via a small hole in a sterilized glass coverslip (18 mm diameter). Embryos were then incubated for 2 hours at which time 100 µL of 0.5% Evan's Blue, 5% BSA in PBS was injected and embryos were further incubated for 60 minutes. After incubation, the embryos were perfused with saline. The tissue underlying the coverslip was removed after the treatment period and blotted dry, weighed, homogenized and incubated in 200 µL of 100% formamide to release the extravasated dye. Tissue samples were homogenized for 30 sec and then incubated for 48 hr at 38°C. The samples were centrifuged (14000 g for 10 minutes) and 175 µL of supernatant quantified spectrophotometrically against a formamide blank at 620 nm. Vascular permeability index was calculated as dye concentration in treated tissue sections/dye concentration in matched vehicle (PBS) treated samples. For rat studies, Evans Blue dye solution (10 ml/kg body weight, 0.5% Evans blue (w/v) in endotoxin-free PBS) was injected intravenously. Ten minutes after injection, each rat (n = 5) was injected intradermally with 25 µL of PBS into the left ear and 0.15nmoles of reagent (∼25 µL) into the right ear. Thirty minutes later, rats were anesthetized, their ears photographed, and then perfused with 100 mL PBS thru a ventricular infusion to remove free intravascular dye. The ears were removed, the area of extravasation cut out with a biopsy punch (8 mm wide), and then the tissue was weighed and subsequently placed into 1 mL of formamide for elution of the Evans Blue dye at 60°C over the course of 48 hours. The amount of extravasated dye was then determined spectrophotometrically as described above. The absolute amount of dye was determined using a standard curve.

### Cell Lines and Tumor Xenografts

Epidermoid carcinoma (HEp3) or breast cancer (MDA-MB435) cells expressing green fluorescent protein (GFP) were maintained as described previously [37]. For imaging studies involving xenograft tumors, day 10 chicken embryos had 0.1–0.5×10^6^ tumor cells in serum free media applied directly to a section of the CAM surface that had been lightly abraded with a piece of filter paper. For embryos being prepared for intravital imaging, sterilized coverslips were applied on top of the tumor 24 hr post tumor cell application.

### Intravital imaging

Fertilized Dekalb White chicken eggs were received from Cox Bros Poultry Farm (Maitland, NS) and incubated in a humidified chamber at 38°C. At day 4, embryos were removed from their shells using a Dremel tool with a cutting wheel and maintained under shell-less conditions, in a covered dish in a humidified air incubator at 38°C and 60% humidity as previously described [38], [39]. On day 10 of development, chicken embryos were injected with 50 µg of fluorescein isothiocyanate (FITC)-dextran (2 MDa) (Ex/Em 494 nm/521 nm), tetramethyl rhodamine isothiocyanate (TRITC)-dextran (158 kDa) (Ex/Em 550 nm/573 nm) or doxorubicin hydrochloride (Sigma) (Ex/Em 470 nm/556 nm) using a glass microinjection needle into a small venule in the CAM. Injected volumes were maintained at 50 µL. The natural fluorescence of doxorubicin was captured using the same filters (Ex: BP 550/25 (HE), Em: BP 605/70 (HE)) used for TRITC signal capture. Immediately after injection of the fluorescent reagents, real-time imaging of the CAM was performed using a previously described chick-embryo-imaging unit [39], [40].

### Image capture and processing

Real-time imaging of vascular leak was performed using an upright epifluorescence microscope with a motorized Z stage (AxioImager Z1, Carl Zeiss, Thornwood, NY) controlled by Volocity software (Improvision, Lexington, MA). A four dimensional image series was collected by capturing a mosaic of 3D image stacks at distinct time-points from regions of interest within the CAM. Specifically, a 150 nm image stack mosaic was captured with a 15 nm step size every 15 min for 3–6 hours (13–25 frames). From this raw data at each time point, the image stack was cropped to a 100 nm stack containing the in-focus images of the tumor. This was then flattened into a maximum intensity projection for the majority of the analyses using Volocity software (Improvision, Lexington, MA).

Time 0 was defined as the time of the first image capture, 5 minutes after injection of the fluorescent dextran mixture. The captured images were corrected for drift and rotation using the Stackreg plugin (Biomedical Imaging Group, http://bigwww.epfl.ch/) of ImageJ (NIH, Rasband, W.S., ImageJ, National Institutes of Health, Bethesda, Maryland, USA, http://rsb.info.nih.gov/ij/, 1997–2004). To generate the time-dependent changes in fluorescence localization in the CAM, the time 0 image stack was subtracted from the subsequent time-points using the Image Calculator function within Image J, hence time 0 intensity was set at 0. For the generation of surface plots, end point images were processed using the Interactive 3D Surface Plot plug-in for ImageJ (Internationale Medieninformatik, Berlin, Germany, http://rsbweb.nih.gov/ij/plugins/surface-plot-3d.html). Relative intensities in the surface plots were qualified using a spectrum LUT normalized from standard 255 levels to 100 levels for ease of interpretation. The pseudo colored spectrum LUT is based on 255 shades of grey in which a Value 0 = black and a Value 255 = white.

## Results

### The Miles assay predictably measures vascular permeability changes in the CAM

**Figure 1:**
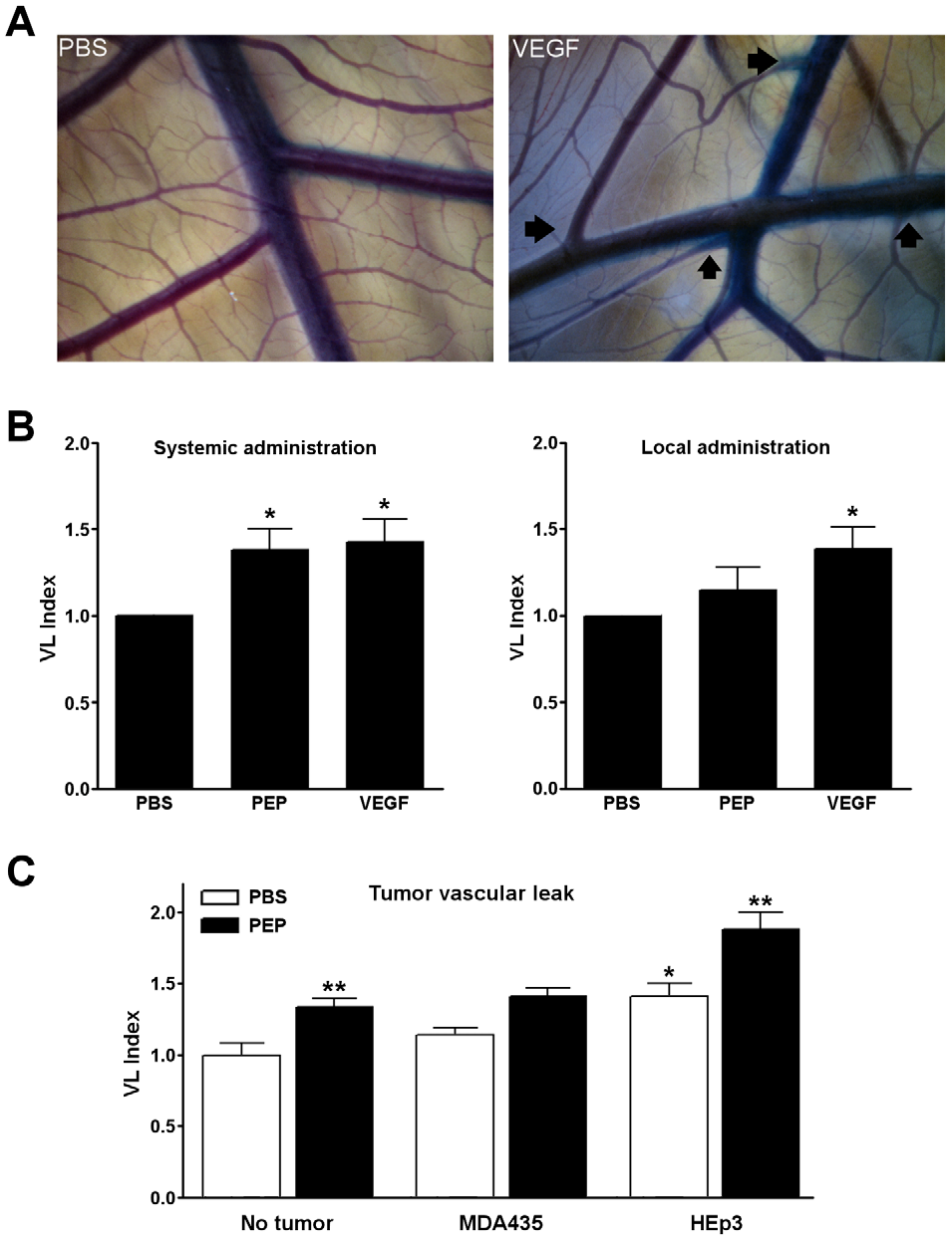
The Miles assay measures vascular permeability changes in the CAM. **A.** Bright field images of CAM vasculature following injection of Evan's blue dye subsequent to the systemic administration of PBS (left panel) or VEGF (right panel). Arrows indicate areas of visible vascular leak. **B.** When VEGF or PEP is injected intravenously distal to the site of analysis, a significant level of vascular permeability is observed in the CAM (left). Topically administered VEGF but not PEP induces a significant level of vascular permeability (right). **C.** Vascular permeability changes in the CAM were evaluated in the presence of human tumor xenografts. Increased vascular permeability was observed at the tumor site, particularly in HEp3 tumors. Systemically administered PEP (0.1 nM) further increases vascular permeability. Data are presented as Mean +/− SEM, n>15 for each group. * indicates statistical significance, p<0.05, ** p<0.01.

To validate the shell-less chicken embryo as a suitable model for vascular leak analysis, we performed an adapted Miles assay to assess the impact of permeability enhancing factors VEGF and PEP. PEP is a 37 amino acid peptide fragment of IL-2 that possesses the vasopermeability activity of intact IL-2 but lacks its cytokine activity [36]. A dose response curve for PEP indicated that maximal dye leakage from vessels resulted from 0.1 nM PEP treatment (data not shown), and this concentration was used in the subsequent experiments. A VEGF concentration of 200 nM was selected for experiments, since this concentration induces significant vascular leakage in rodent models [41], [42], [43]. VEGF, PEP or PBS control was injected into a CAM vein distal to the site of analysis or applied topically to a defined area of the CAM and the embryo was incubated for 2 hours. This was followed by a systemic injection of 0.5% Evan's Blue, 5% BSA in PBS and embryos were further incubated for 60 minutes before processing. The relative vascular leak in PBS, VEGF or PEP-treated vessels was determined in CAMs of day 15 chicken embryos (n≥15 in all cases). Embryos treated with PBS only showed no visible leakage of Evan's Blue dye (Figure 1A, left panel). Leakage of dye was visibly increased in the CAM following injection of VEGF relative to control (Figure 1A, right panel). Systemic injections of VEGF or PEP induced significant increases in CAM vascular permeability, * p<0.05, compared to PBS vehicle controls (Figure 1B). When these agents were applied locally to the surface of the CAM, a significant vascular leak was observed for VEGF, p<0.05 but not PEP (Figure 1B). This may be due to a reduced ability of PEP to diffuse into the tissue. Following a five day growth period of human tumor xenografts MDA-MB435 or HEp3 on the CAM surface (n>22 in all cases), vascular permeability was measured following systemic injection of either PBS or PEP (0.1 nM). The presence of Hep3 tumors, but not MDA-MB435 tumors, resulted in significant vascular permeability when compared to CAM with no tumor xenografts. Injection of PEP increased vascular permeability in the CAM as expected. However, PEP injection significantly amplified vascular permeability in the CAM and in CAMs with HEp3 xenografts (Figure 1C). These findings were consistent with the increase in vascular leak observed in rat models following the administration of PEP (Figure S1). We conclude that the chicken embryo model responds predictably to the systemic administration of permeability factors VEGF and PEP or to in situ factors secreted by a tumor xenograft and the resulting changes in vascular leak are quantifiable using the Miles assay.

### Changes in vascular permeability can be visualized using intravital imaging

**Figure 2:**
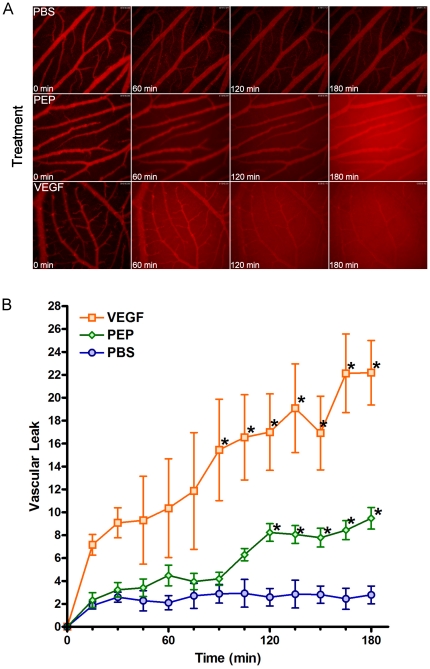
Intravital imaging assesses real-time changes in vascular permeability induced by VEGF and PEP. **A.** A series of representative images from intravital imaging experiments is shown. An accumulation of fluorescence outside the vasculature over time is seen in those embryos treated with VEGF or PEP compared to PBS. **B.** Images were captured and quantified every 15 minutes over a period of 3 hours to evaluate the extent of vascular leak. Vascular leak values were generated by subtracting time 0 values from subsequent time points. Asterisks indicate significant leak of dextran from the vasculature (2-way ANOVA, p<0.05 followed by Bonferroni post-tests, (p<0.05)) comparing either PBS vs VEGF, or PBS vs PEP at each time point.

The *ex ovo* chicken embryo is an emerging platform for intravital imaging of angiogenesis and the tumor microenvironment [39]. We have previously demonstrated that dye-labeled dextrans are not particularly useful for the long-term visualization of vasculature *in vivo*, as they leak progressively into the interstitium [44], [45]. As indicators of changes in vascular permeability and tumor perfusion, however, dextrans are potentially very useful, since they mimic endogenous proteins by extravasating from vessels predictably based on size [20]. To assess this, a 158 kDa TRITC-dextran was selected for its similar size to immunoglobulins, which passively extravasate through blood vessel walls [20]. Based on the published pharmacokinetics of fluorescently labeled dextrans [20], we selected a particle size that should extravasate from the vasculature at a slow but measurable rate, that might be influenced by vascular permeability factors. Intravital imaging following systemic injection of VEGF or PEP revealed a significant induction of vascular leak, manifested by a decrease in TRITC fluorescence in the vessels over time, and a simultaneous increase of signal in the surrounding tissues (Figure 2A). Extravasation of the 158 kDa TRITC-dextran was detectable within 30 minutes after PEP or VEGF treatment (Figure 2B and Video S1). Significant levels of vascular leak were not detected in PBS-treated controls over 3 hours of imaging (Figure 2B). This basic approach allows for real-time visualization of the vascular network and the dynamic measurement of temporal changes in vascular permeability.

### Measuring the structural integrity and permeability of tumor vasculature

**Figure 3:**
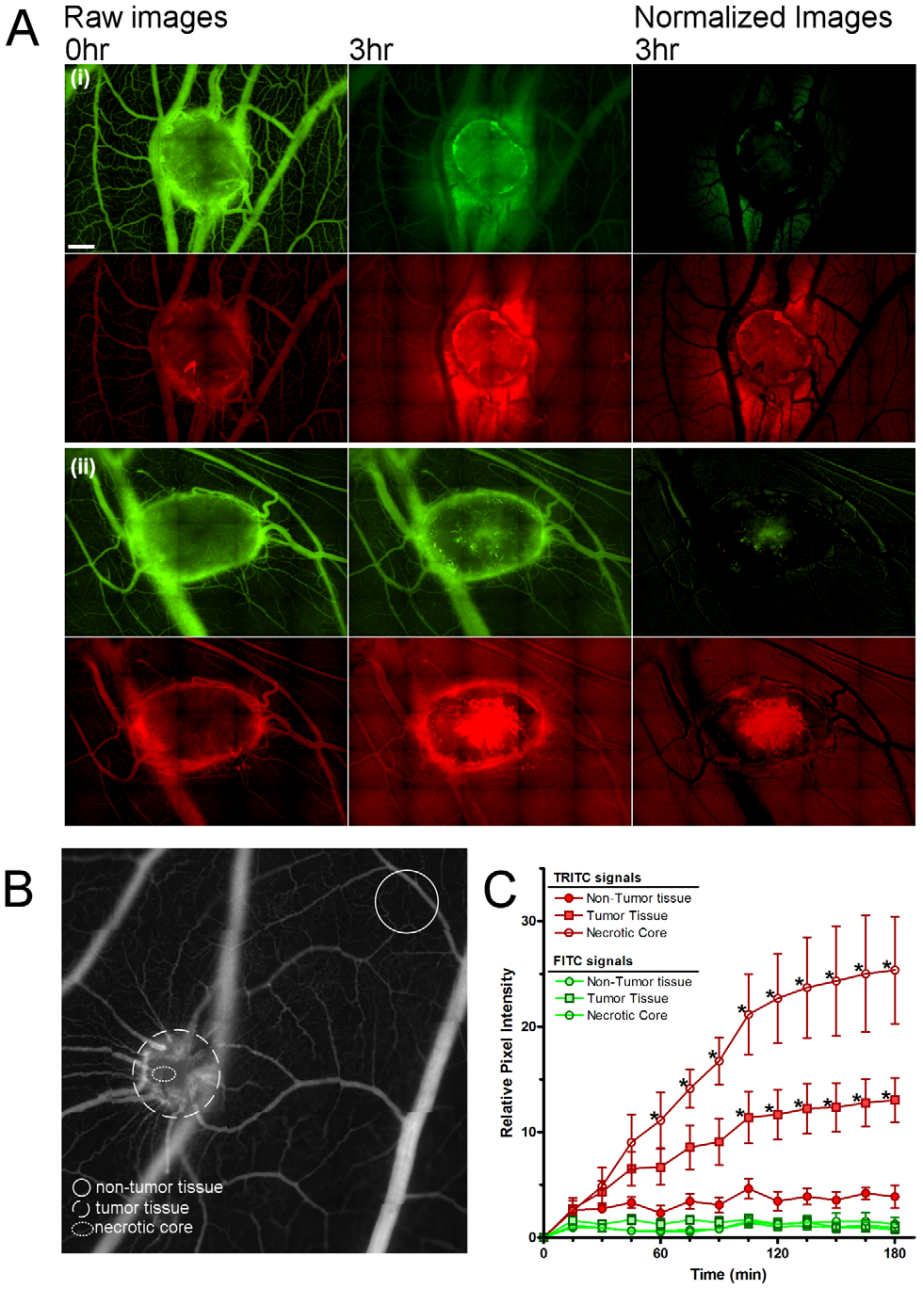
Assessment of regional permeability and vascular integrity in human tumors. **A.** Representative fluorescence micrographs from two human HEp3 tumors displaying peri-tumoral (i) and tumor core (ii) vascular leak are shown with the 2000 kDa FITC-dextran (green) and 158 kDa TRITC-dextran (red). The normalized images were generated by subtracting the 0 hour image from the 3 hour image, and represent the net vascular leak. Tumor induced vascular leak is localized primarily to the tumor and especially to the central, necrotic core of the tumor. **B.** Areas utilized for regional vascular leak analyses are delineated. The solid circle represents an area of non-tumor tissue; the dashed circle denotes tumor and the dotted line indicates the avascular necrotic core. **C.** Quantitation of leak of large (green) and small (red) dextrans is shown for non-tumor tissue, the entire tumor and the core of the tumor. The relative leak of both dextrans was normalized to time zero; n = 6 for each analysis. Two-way ANOVA, (p<0.05) followed by Bonferroni post-tests, (p<0.05) was used to assess significant leak of the TRITC-dextran of either tumor versus non-tumour tissue, and necrotic core versus non-tumour tissue at each timepoint. Timepoints that demonstrated significance are indicated by an asterisk.

The tumor vasculature is heterogeneous, consisting of irregularly formed vessels that are both leaky and often fenestrated [46]. The extravasation of plasma proteins would be expected to occur via enhanced vascular permeability and leak through discontinuous vessel walls [47]. In order to distinguish between these two phenomena, we visualized co-injected dextrans of 2000 kDa or 158 kDa simultaneously. The 2000 kDa fluorescent dextran was selected to mimic large molecular weight blood components, such as LDL (2500–3500 kDa) and VLDL (10–80×10^4^ kDa), which are largely retained by structurally intact vasculature [20]. We hypothesized that structurally intact vasculature would retain the 2000 kDa FITC-dextran, and thus it could be used to define the functional vessel framework in a given region of interest. Furthermore, we surmised that the change in the ratio of the 158 kDa dextran to the 2000 kDa dextran could provide a quantitative measure of vascular permeability changes over time at the tumor site. To this end, 158 kDa TRITC-dextran and 2000 kDa FITC-dextran were systemically co-injected into CAMs bearing human tumor xenografts with an average weight of 25–50 mg and a diameter range of 3–8 mm. At the tumor site, significant vascular leak of the smaller 158 kDa TRITC dextran was detected after 45 minutes, and after 180 minutes was 3.5-fold greater in the tumor than in normal tissue distal to the tumor site. Vascular leak of either the large or small dextrans was nominal in the absence of a tumor. The increased vascular leak seen in tumor-bearing CAMs was greatest in regions immediately surrounding or within the tumor (see Video S2 and S3). By imaging in real time the extravasation of these two dextran populations, it is possible to simultaneously monitor both structural and functional aspects of the vasculature, and to precisely evaluate regional differences in vascular permeability (Figure 3). Comparison of stitched images (30–40 frames) from the tumor versus normal tissue shows significantly more leak in the necrotic tumor core versus the non-tumor tissue after 60 minutes (2-way ANOVA, p<0.05 and Bonferroni post-tests for each time point, p<0.05). Necrotic core vascular leak of the 158 kDa TRITC-dextran was more than 6-fold greater than leak in tissue distant to the tumor after 180 minutes. Comparison across tissue in the tumor shows significantly more leak was detected in the necrotic core versus the tumor after 120 minutes (2-way ANOVA, p<0.05 and Bonferroni post-tests for each time point, P<0.05). Indeed, significantly increased extravasation of the 158 kDa dextran was seen at the tumor core compared to the entire tumor or to normal vasculature distal from the tumor (Figure 3C). Because the tumor xenografts in this assay develop on top of the CAM, there was some concern that the environment may be atypically oxygen rich. We found that this was unlikely to be the case, as tumors grown in the CAM underneath a glass coverslip had equivalent vascular leak levels as those exposed to the air.

### Increased vascular permeability enhances drug delivery to tumor sites

**Figure 4:**
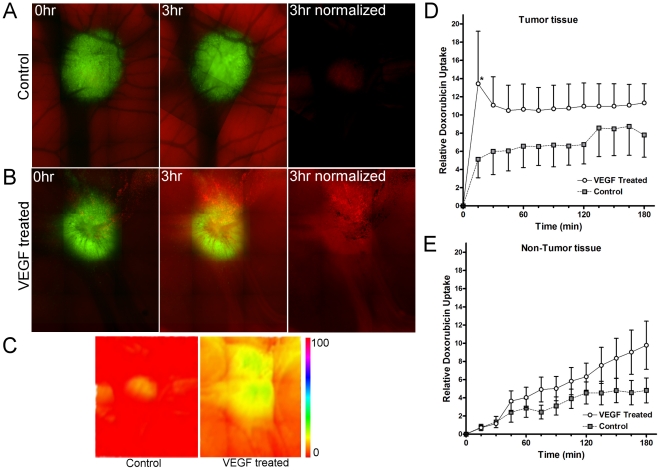
Increasing vascular permeability enhances the accumulation of doxorubicin into the tumor. Doxorubicin was injected intravenously subsequent to administration of PBS or VEGF and its uptake at the tumor site in real time was estimated using its natural fluorescence. **A–B.** Representative images of doxorubicin uptake over time, tumors (green) and doxorubicin uptake (red) are shown. **C.** Heat map of doxorubicin uptake after 3 hours in control and VEGF-treated tumors. **D.** Graph showing relative uptake of doxorubicin in the tumor in the presence or absence of systemic VEGF treatment. **E.** Graph showing relative uptake of doxorubicin in the normal tissues distal to the tumor in the presence or absence of systemic VEGF treatment. N = 4 per treatment; data were analyzed by 2 way ANOVA.

Given that the vascular permeability in tumors is elevated, and that the vasoactive agents VEGF and PEP enhance vascular permeability, we hypothesized that the delivery of chemotherapeutic drugs to tumor sites could be improved using these agents. To test this, we measured the delivery of chemotherapeutic agents to tumor sites in real time using intravital imaging in the presence and absence of VEGF (Figure 4A–C). Doxorubicin was detected by its natural fluorescence using intravital imaging. When doxorubicin was injected systemically into embryos bearing HEp3-GFP tumor xenografts, its uptake into tumors in the absence of VEGF increases over 60 minutes, reaching maximum levels after 2 hours (Figure 4D). When 200 nM VEGF was co-administered with doxorubicin, doxorubicin uptake by tumor tissues was enhanced over 4× within 15 minutes (p<0.05, 2-way ANOVA, Bonferroni post test) compared to control levels (Figure 4E). At 60 minutes, doxorubicin uptake in the presence of VEGF was approximately twice that of the controls. Doxorubicin uptake into normal tissues was also increased by co-administration of VEGF, but to a lesser extent (40%) than at the tumor site. Thus, the delivery of doxorubicin to tumor xenografts was significantly and selectively enhanced by the transient systemic administration of VEGF.

## Discussion

Here, we present a novel approach to visualize and quantitate hemodynamics and vascular leak in tumors. In contrast to traditional, endpoint analysis methods, real-time intravital imaging is sensitive to changes in both permeability and vessel integrity, and can effectively track rapid and dynamic changes in vascular permeability. We demonstrate that vascular permeability is increased in xenograft tumors compared to distal normal tissues, and that it can be further enhanced by VEGF and the PEP fragment of IL-2. Utilizing standard epifluorescence microscopy, we could also monitor localization of the chemotherapeutic, doxorubicin, which is a naturally fluorescent DNA intercalating agent, using this approach. We show that the uptake of systemically administered doxorubicin in xenograft tumors is enhanced by co-administered VEGF, suggesting that transiently increasing the vascular leak in tumors using adjuvant therapies can improve the uptake of chemotherapy at the tumor site.

As an alternative to the model we present here, vascular dynamics can be visualized in rodent models with the use of surgically placed skin flaps. Skin flaps have been used to estimate the vascular leakage of florescent-labeled particles under various conditions in tumors in rats [48] hamsters [49], and mice [9], [20], [50] and to measure vessel regeneration during wound healing [51]. Imaging through skin flaps can predict drug localization [20] and the influence of treatments on hemodynamics and vascular permeability [9], [48], [49], [50]. However, imaging protocols in rodents can be complicated. Creating the necessary skin flaps requires microsurgical implantation of a frame in anesthetized animals to provide a viewable imaging area. This nontrivial procedure can complicate vascular dynamics and permeability around the viewing area by inducing inflammation. By comparison, the chick model described here is relatively easy to maintain while the thin, vascular and transparent nature of the CAM is amenable to imaging without surgical intervention. Despite the chick CAM's simplicity, our biological findings are consistent with those reported in more complex models. Therefore, this work further validates the chick CAM's use as a tumor model and suggests on its potential use for semi-high throughput imaging and screening analysis that should facilitate and compliment the use of more complex mammalian models.

The chick CAM responds predictably to permeabilization factors and supports the growth of human tumor xenografts. While increased vascular permeability induced by VEGF and PEP occurred within minutes, imaging time courses of up to 72 hours, can be accommodated in the *ex ovo* chicken embryo model [39]. Although fluorescent dextrans were used in the methods described here, the CAM model will likely accommodate alternate molecules to further expand its utility, such as labeled immunoglobulins or LDL. These considerations along with the conservation of the key chicken and human angiogenesis factors make it a useful model to understand angiogenesis [52], [53], drug targeting [54], [55], response to therapeutics [32] and vascular permeability.

Selective modification of the tumor vasculature is emerging as a powerful means to enhance drug delivery and ultimately efficacy. Common vasoactive agents used in oncology follow two principle approaches; perturbation of the tumor vasculature by vascular disrupting agents (VDAs) or normalization of the tumor vasculature by anti-angiogenic agents. For example, the tubulin-binding agent, combretastatin-serine (AVE8062) is a small molecular weight VDA that causes a rapid and extensive shutdown of established tumor vasculature. Prior dosing with AVE8062 can therapeutically synergize with docetaxel, oxaliplatin or cisplatin [56], [57]. Therapies to normalize the tumor vasculature, as described by Jain [58], suggest that following disruption of the immature vessels, the mature tumor vasculature becomes strengthened and hence more susceptible to drug therapy. Current strategies typically include the use of VEGF inhibitors such as bevacizumab or anti-VEGF antibody, which has shown benefits in animal models and patients [7], [59]. Tong and coworkers showed decreased interstitial hypertension caused by targeting VEGF produced a morphologically and functionally “normalized” vascular network resulting in pressure gradients favoring extravasation and hence improving drug penetration in tumors [7].

Increasing the uptake of co-administered chemotherapies by overcoming the high interstitial fluid pressures in the tumor microenvironment has previously been accomplished through inhibition of the PDGF receptor with imatinib [60], [61], remodeling of the extracellular matrix using collagenase and hyaluronidase [8], [62], vascular normalization using anti-VEGF antibodies [7] and targeted vasopermeation using PEP [63] (for review, see Cairns et al., 2006 [64]). While the overall goal is the same, evidence suggests that similar strategies can have markedly different consequences. Tong et al. [7] suggest that drug uptake is improved at the tumor site during vascular normalization because a pressure gradient is briefly formed across the vessel walls in tumors. This gradient dissipates rapidly, however, against the high interstitial fluid pressure in the tumor. They suggest that this short time window should be sufficient to improve drug uptake. This contrasts with observations by Khawli et al. [63], who administer PEP immunoconjugates two hours prior to chemotherapy to achieve an optimal increase in drug uptake at the tumor site. Our data indicate that VEGF and PEP rapidly increase vascular leak, with measurable increases in dextran efflux over controls that are apparent within 15 minutes and continue to increase for 3 hours. Interestingly, VEGF treatment resulted in a different dynamic in doxorubicin uptake, with an initial spike in doxorubicin accumulation in tumor tissue that was maintained at a steady level throughout the 3 hours of analysis. The subtle difference in dextran versus doxorubicin accumulation may result from size differences between the dextran and doxorubicin molecules. The larger dextran molecule likely requires a greater change in permeability and thus responds more slowly than the smaller doxorubicin molecule. Clearly, understanding the unique features of tumor blood dynamics and vascular leak will help tease out these subtle, but consequential affects to appropriately focus chemotherapeutic delivery systems.

Given the dynamic interplay of vascular signaling factors and their individual roles in the modulation of vascular permeability, it is difficult to predict the impact of adjuvant permeability enhancement agents on drug uptake at the tumor site. The approach and the model presented here offer a powerful tool to investigate mechanisms of vasopermeability *in vivo* and to screen the most appropriate strategies for improving drug uptake.

## Supporting Information

Figure S1 **Comparison to rodent ear model of vascular permeability.** Injection of PEP (0.15 nmoles) into the ears of rats (n = 5) induces significant levels of vascular permeability similar to CAM data in Figure 1. As an internal control, PBS was injected into the corresponding left ear. The ratio of vascular leakage seen for the reagent ear (right ear) divided by the value for the PBS ear (left ear) was graphed as a VL index. Data are presented as Mean +/− SEM. * indicates statistical significance, p<0.05.
(TIF)

Video S1 **Intravital imaging assesses real-time changes in vascular permeability.** Representative intravital imaging experiments representing the extravasation of 158 kDa dextran are shown for PBS, VEGF and PEP-treated embryos over 3 hours. Top panels represent the raw imaging data; bottom panels are normalized to the first time point to denote leaked dextran.
(AVI)

Video S2 **Intravital imaging of vascular permeability in HEp3 tumor.** Intravital imaging experiments of 158 kDa (red) and 2000 kDa (green) dextran extravasation over time are shown for human epidermoid carcinoma (HEp3) tumors established in the CAM. Raw and normalized data are shown.
(AVI)

Video S3 **Intravital imaging of vascular permeability in MDA-MB435 tumor.** Intravital imaging experiments of 158 kDa (red) and 2000 kDa (green) dextran extravasation over time are shown for human breast carcinoma (MDA-MB435) tumors established in the CAM. Raw and normalized data are shown.
(AVI)
