# Supplementary figures and images for: Real-Time Visualization and Quantitation of Vascular Permeability In Vivo: Implications for Drug Delivery
Source: PLoS One. 2012 Mar 29;7(3):e33760. doi: 10.1371/journal.pone.0033760 (PMC3315578; doi:10.1371/journal.pone.0033760)

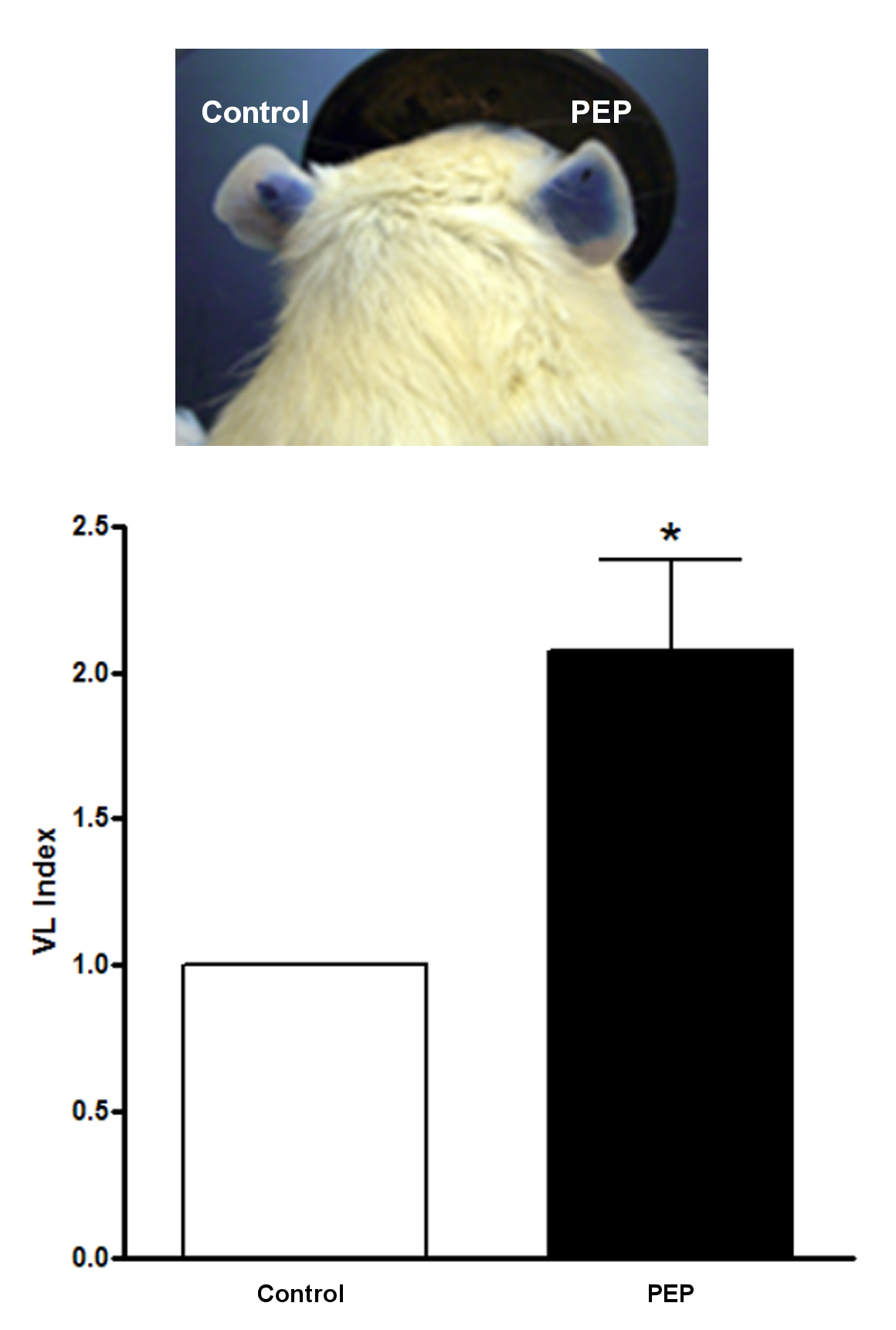

Supplement: Figure S1 — Comparison to rodent ear model of vascular permeability. Injection of PEP (0.15 nmoles) into the ears of rats (n = 5) induces significant levels of vascular permeability similar to CAM data in Figure 1. As an internal control, PBS was injected into the corresponding left ear. The ratio of vascular leakage seen for the reagent ear (right ear) divided by the value for the PBS ear (left ear) was graphed as a VL index. Data are presented as Mean +/− SEM. * indicates statistical significance, p<0.05. (TIF) [file pone.0033760.s001.tif]
